# Cancer Incidence Projections in the United States Between 2015 and 2050

**DOI:** 10.5888/pcd18.210006

**Published:** 2021-06-10

**Authors:** Hannah K. Weir, Trevor D. Thompson, Sherri L. Stewart, Mary C. White

**Affiliations:** 1 Division of Cancer Prevention and Control, Centers for Disease Control and Prevention, Atlanta, Georgia

## Abstract

**Introduction:**

The number of adults entering the age groups at greatest risk for being diagnosed with cancer is increasing. Projecting cancer incidence can help the cancer control community plan and evaluate prevention strategies aimed at reducing the growing number of cancer cases.

**Methods:**

We used data from the Surveillance, Epidemiology, and End Results Program and the US Census Bureau to estimate average, annual, age-standardized cancer incidence rates and case counts (for all sites combined and top 22 invasive cancers) in the US for 2015 and to project cancer rates and counts to 2050. We used age, period, and cohort models to inform projections.

**Results:**

Between 2015 and 2050, we predict the overall age-standardized incidence rate (proxy for population risk for being diagnosed with cancer) to stabilize in women (1%) and decrease in men (−9%). Cancers with the largest change in risk include a 34% reduction for lung and bronchus and a 32% increase for corpus uterine (32%). Because of the growth and aging of the US population, we predict that the annual number of cancer cases will increase 49%, from 1,534,500 in 2015 to 2,286,300 in 2050, with the largest percentage increase among adults aged ≥75 years. Cancers with the largest projected absolute increase include female breast, colon and rectum, and prostate.

**Discussion:**

By 2050, we predict the total number of incident cases to increase by almost 50% as a result of the growth and aging of the US population. A greater emphasis on cancer risk reduction is needed to counter these trends.

**Summary**

**What is already known on this topic?**

In the United States, the number of adults entering the age groups at greatest risk for being diagnosed with cancer is increasing.

**What is added by this report?**

Between 2015 and 2050, we predict the total number of cancer cases to increase by almost 50% as a result of the growth and aging of the US population. The largest increase is anticipated in adults aged ≥75 years.

**What are the implications for public health practice?**

Projecting cancer cases can help the public health community plan and evaluate community intervention strategies aimed at reducing the growing number of cancer cases by reducing cancer risk across the lifespan.

## Introduction

In the US, cancer is the leading cause of death in midlife and may soon become the leading cause of death overall as the number of people diagnosed with and dying from cancer continues to increase (1,2). Paradoxically, over the past several decades the overall age-standardized cancer incidence rates have stabilized and death rates have declined steadily. The age-standardized rate can be used as a proxy for the population’s risk of being diagnosed with or dying from cancer and is useful for comparing risk between populations or over time within a population. However, the age-standardized rate effectively removes the underlying influence of demographic changes in the population. The risk of being diagnosed with cancer generally increases with age, and over this period the US population has grown, particularly in the older age groups (2,3). Thus, the increase in the number of incident cases and deaths reflects, to a large extent, the impact of a growing and aging population. This demographic trend is expected to continue as a larger proportion of the Baby Boom and Gen X cohorts survive to older ages compared with earlier generations and enter the age groups most at risk for a cancer diagnosis.

Trends in cancer incidence rates (population risk) and projections of population growth and age structure have been used to predict cancer incidence including in the US (4), Canada (5), England (6), the Nordic countries (7), and for world regions broadly (8). Predicting the growth in the number of incident cases in the US can help health planners and policy makers anticipate the resources needed to diagnose, treat, and care for future cancer patients and cancer survivors. Cancer-specific projections can also help the public health community to plan and evaluate risk reduction strategies and alert researchers to early changes in population risk.

In this study, we used data from the National Cancer Institute’s Surveillance, Epidemiology, and End Results (SEER) Program to estimate nationwide, age-standardized, 5-year average annual cancer incidence rates and case counts (all sites and top 22 cancers) for the US population for 2015 and to project rates and counts to 2050.

## Methods

### Data sources

We obtained data for patients diagnosed with invasive cancer from 1996 through 2015 from the SEER Program, which covered approximately 14% of the US population (9). The file included in situ bladder cancer cases because these cancers are considered invasive for the purpose of incidence reporting (10). Population estimates used as rate denominators were a modification of annual county age- and sex-specific population estimates produced by the US Census Bureau’s Population Estimates Program, in collaboration with the Centers for Disease Control and Prevention’s (CDC’s) National Center for Health Statistics and with support from the National Cancer Institute (11). We obtained population projections of the resident population (Middle Series) by age and sex from 2016 through 2050 from the US Census Bureau’s Population Projections Program (12).

### Analytic methods

We used SEER*stat to calculate age-specific and age-standardized rates for cancer patients of all ages who were diagnosed with invasive cancer (other than nonmelanoma skin cancer) from 1996 through 2015. All invasive cancers were selected and grouped according to the top 22 cancers and all other remaining sites combined among men and women. We estimated nationwide, annual incident counts for 2015 by applying 5-year age-specific incidence rates (2011–2015) to the 2011–2015 US population estimates and dividing by 5. Similarly, projections for 2050 were calculated by annualizing rates and population projections for the 2046–2050 period. Methods for projecting cancer incident cases in the US have been published previously (4,5). Briefly, to project cancer incidence rates from 2016 through 2050, we used NORDPRED software, available from the Cancer Registry of Norway website (www.kreftregisteret.no/en/Research/Projects/Nordpred/Nordpred-software/) (7). The program used age–period–cohort regression models with input data aggregated into four 5-year calendar periods (1996–2000, 2001–2005, 2006–2010, 2011–2015) and 15 age groups (15–19 years through ≥85 years). Separate models were fit for each cancer site by sex and all races combined: R_ap_ = (A_a_ + D•p + P_p_ + C_c_)^5^ in which the dependent variable R_ap_ is the incidence rate in age group a in calendar period p. A_a_ is the age component for age group a, D is the drift parameter (the common linear effect of both calendar period and birth cohort), P_p_ is the nonlinear period component of period p, and C_c_ is the nonlinear cohort component of cohort c. When using the regression models as the basis for projected rates for each cancer site and sex group, the starting age criterion was that each age group contain 10 or more cases. Projections for age groups below that starting criterion were based on the average rates from the past 10 years. Separate models were fit for each cancer site by sex. To offset exponential increases or decreases in incidence rates, we used the power-5 link function. Assuming that trends are not likely to continue indefinitely, the drift component in the model was reduced by 25% in the second calendar period, by 50% in the third calendar period, and by 75% in the fourth and fifth periods. These modifications have been shown empirically to improve predictions (7).

We based projections on 20 years of data (1996–2015) unless significant curvature in the trend was found over time. When curvature occurred, the linear drift component was based on the most recent 10-year period. Projections for all sites combined were summed estimates for the cancer sites categories, including other cancer sites combined. For thyroid cancer, we used a modified approach to account for recent concerns that overdiagnosis may inflate projections (13). We based projections for thyroid cancers on age-specific rates for thyroid cancer diagnosed from 2011 through 2015 because recent thyroid incidence rates are no longer increasing (14). For female breast and prostate cancer, we used a modified approach to account for breast cancer incidence decreases in the early 2000s attributed to a reduction in the use of hormone replacement therapy and fluctuations in prostate cancer incidence related to the use of the prostate-specific antigen test (15,16). For these cancers, we had the trends taper off sooner by applying 25%, 50%, and 75% reductions in the first 3 calendar periods and truncating the trends (ie, 100% reduction) in the fourth and fifth periods.

NordPred provides projections for up to five 5-year periods; thus, age-specific incidence rates were projected for the 5-year calendar periods 2016–2020, 2021–2025, 2026–2030, 2031–2035, and 2036–2040. Projections for 2041–2045 and 2046–2050 were generated by applying the 2036–2040 age-specific incidence rates to corresponding population projections because the greatest driver in overall cancer incident cases has been the growth and aging of the US population (4).

We calculated the absolute and relative difference between estimated 2015 and projected 2050 age-standardized incidence rates and case counts. Annual estimated and projected incident cases and absolute differences were rounded to the nearest 100th for presentation in tables.

## Results

**Figure 1:**
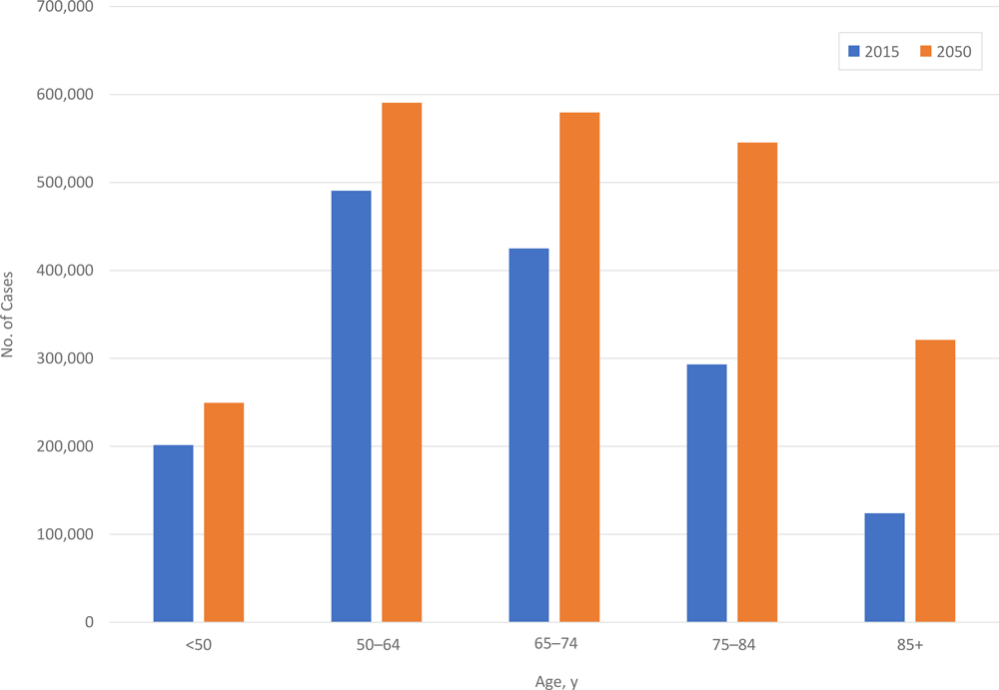
Distribution of estimated 2015 and projected 2050 average annual cancer cases (all sites combined), by age group, United States. Numbers may not sum to total because of rounding.

Table 1 shows the distribution of estimated and projected annual counts of all cancer incident cases for 2015 and 2050, respectively, by age. The total number of cases is predicted to increase by 49% from 1,534,500 (2015) to 2,286,300 annual cases (2050). In each age group, the total number of cases is predicted to increase. The largest percent increase was projected for adults aged ≥85 years followed by adults aged 75–84 years (Figure 1). In 2015, it is estimated that 842,200 (55%) of cancer patients were diagnosed at aged ≥65 years. In 2050, we predict that 1,446,000 (63%) of all patients diagnosed with cancer will be aged ≥65 years, an increase of 603,800 annual cases from 2015.

**Table 1:** Distribution of Estimated (2015) and Projected (2050) Average^a^ Annual Counts of Cancer Cases (All Sites Combined) and Percentage Change, by Age, United States

Age, y	2013	2048	2013–2048
	No. (%)	No. (%)	No. Change (% Change)
<50	201,500 (13)	249,500 (11)	48,000 (24)
50–64	490,700 (32)	590,700 (26)	100,000 (20)
65–74	425,000 (28)	579,500 (25)	154,500 (36)
75–84	293,200 (19)	545,400 (24)	252,200 (86)
≥85	124,000 (8)	321,100 (14)	197,100 (159)
Total	1,534,500 (100)	2,286,300 (100)	751,900 (49)

a 2015 Estimated counts are average annual counts of cancer incident cases diagnosed 2011–2015. 2050 Projected counts are average annual counts of cancer incident cases projected to be diagnosed 2045–2050.

**Table:** 

Age, y	2015 (n = 1,534,500)	2050 (n = 2,286,300)
	No. (%)	
<50	201,500 (13)	249,500 (11)
50–64	490,700 (32)	590,700 (26)
65–74	425,000 (28)	579,500 (25)
75–84	293,200 (19)	545,400 (24)
≥85	124,000 (8)	321,100 (14)

Table 2 shows estimated 2015 and projected 2050 average annual age-standardized incidence rates and case counts. The top 4 cancers (female breast, prostate, lung and bronchus, and colon and rectum) accounted for 49% of all incident cases in 2015 and are projected to account for 46% of all incident cases in 2050. Cancer sites in which there is projected to be a relative percentage increase of 10% or more in age-standardized rates include female breast, kidney and renal pelvis, corpus and uterus, liver and intrahepatic bile duct, and myeloma. The largest absolute and relative increases in incident cases are expected in female breast (123,900; 52%), prostate (82,300; 43%), colon and rectum (67,900; 50%), and melanoma of the skin (48,000; 63%).

**Table 2:** Estimated (2015) and Projected (2050) Age-Standardized Incidence Rates, Average Annual Case Counts and Percentage Change by Cancer Site

Cancer Site	Sex	Age-Standardized Rates			Average Annual Case Counts			
		2015	2050	% Change	2015	2050	Difference, No.	% Change
All cancer sites	Both	428.9	412.6	−4	1,534,500	2,286,300	751,800	49
All cancer sites	Male	467.1	425.0	–9	766,700	1,149,600	382,900	50
All cancer sites	Female	404.3	407.2	1	767,800	1,136,700	368,900	48
Breast	Female	127.0	139.5	10	238,800	362,700	123,900	52
Prostate	Male	110.9	101.9	–8	193,200	275,500	82,300	43
Lung and bronchus	Both	49.7	32.5	–34	178,100	201,700	23,600	13
Lung and bronchus	Male	57.5	37.1	–35	91,500	106,200	14,700	16
Lung and bronchus	Female	43.9	28.6	–35	86,600	95,500	8,900	10
Colon and rectum	Both	37.9	38.8	3	135,100	203,000	67,900	50
Colon and rectum	Male	43.3	44.5	3	70,200	111,700	41,500	59
Colon and rectum	Female	33.3	33.6	1	64,900	91,300	26,400	41
Melanoma of the skin	Both	21.9	20.9	–5	76,700	124,700	48,000	63
Non-Hodgkin lymphoma	Both	19.5	16.3	–16	68,900	96,100	27,200	39
Urinary bladder	Both	19.0	14.4	–24	68,000	96,700	28,700	42
Kidney and renal pelvis	Both	15.1	17.0	13	54,000	92,100	38,100	71
Corpus and uterus, NOS	Female	26.9	35.5	32	52,800	93,100	40,300	76
Thyroid	Both	14.5	14.4	–1	48,100	60,700	12,600	26
Leukemia	Both	13.8	14.3	4	47,700	80,500	32,800	69
Pancreas	Both	12.5	13.4	7	45,200	84,100	38,900	86
Oral cavity and pharynx	Both	10.9	11.6	7	39,700	63,600	23,900	60
Liver and intrahepatic bile duct	Both	9.2	10.4	14	34,500	65,400	30,900	90
Stomach	Both	7.5	8.0	7	26,700	47,000	20,300	76
Myeloma	Both	6.7	7.6	14	23,900	46,100	22,200	93
Ovary	Female	11.9	11.3	–5	22,500	29,100	6,600	29
Brain and other nervous system	Both	6.2	6.2	–1	21,200	29,000	7,800	37
Esophagus	Both	4.0	3.3	–19	14,700	19,900	5,200	35
Cervix uteri	Female	6.9	5.4	–21	11,400	11,600	200	1
Larynx	Both	2.6	1.8	–31	9,700	10,200	500	6
Hodgkin lymphoma	Both	2.5	2.0	–23	8,200	7,800	−400	−5
Other	Both	33.2	32.6	–2	115,400	185,500	70,100	61

Abbreviation: NOS, not otherwise specified.

Cancer sites projected to have fewer than an additional 10,000 annual incident cases between 2015 and 2050 are cancers of the ovary, brain and nervous system, esophagus, cervix uteri, and larynx. No increase in additional incident cases for Hodgkin lymphoma is predicted. Cancer sites with a predicted relative decrease of 10% or more are lung and bronchus, non-Hodgkin lymphoma, urinary bladder, esophagus, cervix uteri, larynx, and Hodgkin lymphoma.

**Figure 2:**
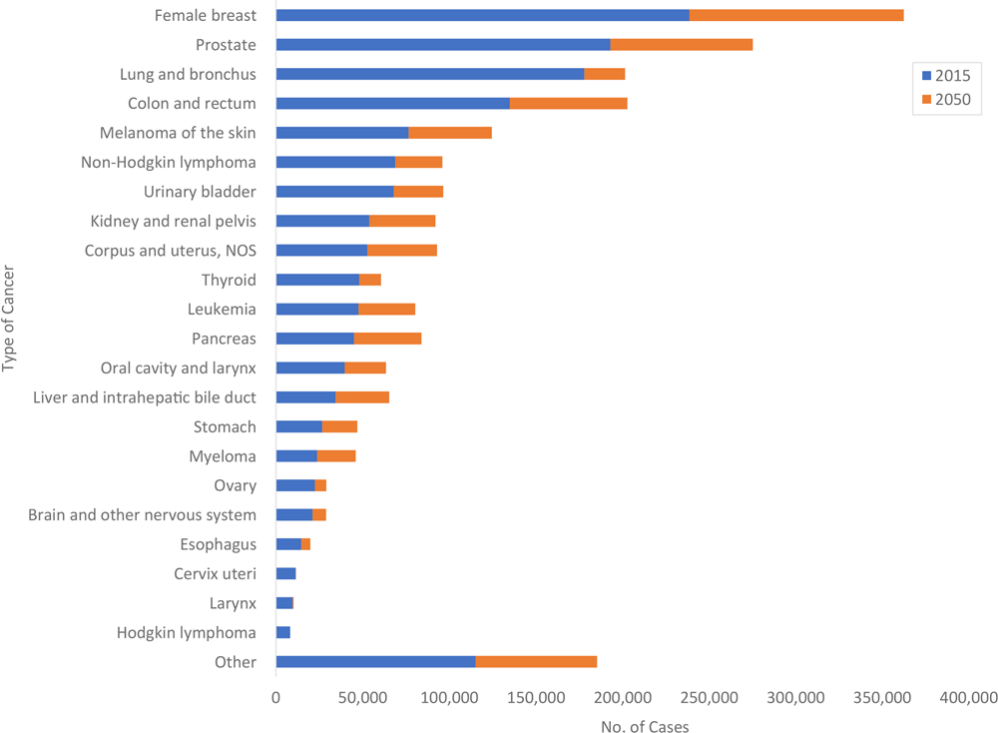
Estimated (2015) cancer cases and projected additional cases (2050) by cancer site, United States. Numbers may not sum to total because of rounding. Abbreviation: NOS, not otherwise specified.

Figure 2 shows the rank order of average annual incident cases estimated to be diagnosed in 2015 and the additional number of annual cases predicted to be diagnosed in 2050. Female breast, prostate, colon and rectum, and lung and bronchus are projected to remain the 4 leading cancers in 2050. In 2015, cancers of the lung and bronchus were estimated to be the third leading cancer diagnosed in men and women followed by colorectal cancers. By 2050, the number of colorectal cancers is predicted to exceed the number of cancers of the lung and bronchus.

**Table:** 

Type of Cancer	2015, n	2050, n
Breast	238,800	123,900
Prostate	193,200	82,300
Lung and bronchus	135,100	67,900
Colon and rectum	178,100	23,600
Melanoma of the skin	76,700	48,000
Non-Hodgkin lymphoma	68,000	28,700
Urinary bladder	68,900	27,200
Kidney and renal pelvis	52,800	40,300
Corpus and uterus, NOS	54,000	38,100
Thyroid	45,200	38,900
Leukemia	47,700	32,800
Pancreas	34,500	30,900
Oral cavity and pharynx	39,700	23,900
Liver and intrahepatic bile duct	48,100	12,600
Stomach	26,700	20,300
Myeloma	23,900	22,200
Ovary	22,500	6,600
Brain and other nervous system	21,200	7,800
Esophagus	14,700	5,200
Cervix uteri	11,400	200
Larynx	9,700	500
Hodgkin lymphoma	8,200	-400
Other	115,400	185,500

## Discussion

Over the next several decades, we predict the total number of cancer incident cases (excluding nonmelanoma skin cancer) to increase by nearly 50%, from 1,534,500 in 2015 to 2,286,300 in 2050. As the size of the US population increases, incident cases are expected to increase in all age groups, but the largest percentage increases will occur among adults aged ≥75 years. Over this period, overall cancer risk is predicted to stabilize in women (1%) and decline slightly (−9%) among men. Thus, the increase in the total number of incident cases will reflect primarily demographic changes related to a growing and aging population.

The demographic components underlying the increase in incident cases are being driven initially by adults born between 1946 and 1964 (the Baby Boom cohort). In 2011, adults in this generation began turning 65 years of age and by 2029, all will be aged 65 years or older. In addition to the increase in the number of incident cases, the number of people living with a history of cancer (cancer survivors) also is expected to increase. Improvements in early detection and cancer treatment of some common cancers resulted in an overall increase in 5-year cancer survival for all cancers combined, from 49% for patients diagnosed in the 1970s to 70% for patients diagnosed in the 2010s (14). An increase in the number of people who receive a cancer diagnosis and high 5-year survival for common cancers like cancers of the female breast and prostate have resulted in an increase in the number of cancer survivors. In 2019, the number of cancer survivors was estimated to be 16.9 million and is projected to reach 22.1 million by 2030 (17). Cancer survivors require ongoing care and surveillance because they are at increased risk for additional cancer diagnoses, as well as other chronic diseases (18). The increase in number of cancer survivors has profound implications for health care and cancer surveillance resource needs in the US, including the need for oncology specialists and certified tumor registrars (19,20). In addition, the costs of cancer care are substantial, increasing, and not sustainable (21,22).

The projections in this study assume that cancer incidence patterns will continue largely unchanged for the next few decades with the 4 leading cancers (female breast, prostate, colon and rectum, and lung and bronchus) accounting for just under 50% of all cancer cases. If the prevalence of causal factors associated with higher cancer risk declined in the population, so too could cancer incidence. Multiple opportunities exist to disrupt the initiation or promotion of different cancers in adults by reducing exposures to carcinogens, promoting social and physical environments that support healthy behaviors, and preventing chronic conditions such as obesity and diabetes (23,24). The Community Guide (www.thecommunityguide.org/) provides recommended community-based strategies to reduce the prevalence of several common behavioral risk factors. Expanded research on environmental cancer and on interventions to reduce inequities in cancer risk could provide additional opportunities to lower cancer incidence in the future (25,26).

A comprehensive cancer control plan can provide a roadmap for public health action to reduce the burden of cancer. Individual state, tribal, and territorial cancer plans in 66 jurisdictions across the US are developed by participants in CDC’s National Comprehensive Cancer Control Program (NCCCP) (27). Program participants can use these findings to prioritize Community Guide and other evidence-based interventions in their plans to help reduce the expected increases in particular cancers, either through the reduction of cancer risk factors or the medical treatment of precancerous conditions, such as the removal of polyps during screening colonoscopy or treatment of cervical lesions detected by Papanicolaou (Pap) tests (27). The NCCCP has historically focused on many of the cancers that are expected to increase in total numbers (female breast, colon and rectum, melanoma, lung and bronchus [through tobacco control], and liver and hepatic duct cancers). In addition to the continued prioritization of these cancers, our data suggest that an expansion of NCCCP’s focus may be warranted in the near term to include reduction and control of cancers of the kidney and renal pelvis and the corpus and uterus. In California, efforts focused on the primary prevention of breast cancer offered an innovative model for integrating scientific evidence on multiple risk factors with community perspectives to develop an action plan (28).

Our analysis has strengths and limitations. Age–period–cohort models identify trends in younger birth cohorts and extrapolate these trends to future older cohorts. These models have been used in many population-based studies, and the methods have been validated using long-term cancer incidence data (7). However, these predictions should be viewed with caution. First, the SEER data covered 14% of the US population, which tended to be more urban and have more foreign-born individuals compared with other parts of the US. As a result, incidence rates based on data from SEER found that 14 areas differed somewhat from data based on National Program of Cancer Registries (NPCR) areas, with prostate incidence higher and lung cancer incidence lower (29). Second, changes in risk factor exposures, screening recommendations, and advances in medical techniques are likely to occur between now and 2050. Finally, population projections are themselves forecasts based on assumptions regarding future births, deaths, and migration and can therefore affect projections of incident counts and rates. Therefore, although our predictions are based on the best available information, they should be updated periodically in consultation with cancer surveillance subject matter experts when combined long-term data from SEER and NPCR become available and as revised population projections become available.

Our projections make it clear that, to mitigate the impact of a growing and aging population, a substantial, robust, and coordinated focus on primary prevention is needed. If these efforts are to have any significant impact on the number of future cancer cases, they must be implemented immediately, owing to the long latency period for many cancers.
